# Correction: Sample preparation considerations for surface and crystalline properties and ecotoxicity of bare and silica-coated magnetite nanoparticles

**DOI:** 10.1039/d1ra90164h

**Published:** 2021-10-28

**Authors:** Lyubov Bondarenko, Vera Terekhova, Anne Kahru, Gulzhian Dzhardimalieva, Elena Kelbysheva, Nataliya Tropskaya, Kamila Kydralieva

**Affiliations:** Moscow Aviation Institute (National Research University) Moscow Russia l.s.bondarenko92@gmail.com; Lomonosov Moscow State University 119991 Moscow Russia; National Institute of Chemical Physics and Biophysics 12618 Tallinn Estonia anne.kahru@kbfi.ee; Institute of Problems of Chemical Physics Chernogolovka Moscow Region Russia; A. N. Nesmeyanov Institute of Organoelement Compounds of the Russian Academy of Sciences Moscow Russia; Sklifosovsky Institute for Emergency Medicine Moscow Russia

## Abstract

Correction for ‘Sample preparation considerations for surface and crystalline properties and ecotoxicity of bare and silica-coated magnetite nanoparticles’ by Lyubov Bondarenko *et al.*, *RSC Adv.*, 2021, **11**, 32227–32235. DOI: 10.1039/D1RA05703K.

The authors regret that the name of one of the authors (Nataliya Tropskaya) was shown incorrectly in the original article. The corrected author list is as shown above.

The authors regret that there was an error in the sentence in line 28 in the right column on page 32227 of the original article. The text originally read, “Not all of these methods cannot be applied to “soft” nanoparticles (such as liposomes, polymeric micelles, dendrimers)^35^ or oxygen-sensitive nanoparticles (NPs) such as iron oxides.” This sentence should read, “Not all of these methods can be applied to “soft” nanoparticles (such as liposomes, polymeric micelles, dendrimers)^35^ or oxygen-sensitive nanoparticles (NPs) such as iron oxides”.

The Royal Society of Chemistry apologises for these errors and any consequent inconvenience to authors and readers.

## Supplementary Material

